# Valorization of iron and high value metals from dust generated during grinding of electroslag remelted ingots

**DOI:** 10.1038/s41598-026-45580-4

**Published:** 2026-04-20

**Authors:** Aadi Anaskure, Adwait Kane, Faratez Siddiqui, Abhiram Puranik, Ravi Prakash Singh

**Affiliations:** 1 Department of Metallurgical Engineering and Materials Science, Indian Institute of Technology Bombay, Mumbai, 400076 India; 2 WMG, University of Warwick, Coventry, CV47AL UK; 3 Saarloha Advanced Materials Private Limited, Pune, 411036 India

**Keywords:** Recycling, Grinding dust, Induction furnace, Briquette, Waste utilisation, Circular economy, Electroslag remelting, Chemistry, Engineering, Environmental sciences, Materials science

## Abstract

Electroslag remelting (ESR) is a secondary refining technique employed to produce ultra-clean specialty steel grades with minimal inclusions and controlled composition. ESR ingots require surface grinding to eliminate defects such as surface cracks, generating fine metallic dust rich in valuable alloying elements. Proper valorization of this dust is important both to mitigate occupational and environmental hazards and to recover commercially significant metals, thereby supporting circular economy objectives within specialty steelmaking. In the present study, dust collected from the surface grinding of SS321 grade ESR ingots was characterised using X-ray fluorescence, X-ray diffraction and wet chemical analysis, confirming a predominantly metallic composition with 74.83 wt% Fe, 10.87 wt% Cr, 7.05 wt% Ni, and 0.65 wt% Mo with no oxide phases detected. An induction furnace-based single-step recovery route was proposed and validated through a pilot-scale trial with a 65 kg charge comprising 30 kg of SS321 electrode material and 35 kg of grinding dust. Elemental mass balance calculations yielded recovery efficiencies of 88.4% for Fe, 85.0% for Cr, 93.1% for Ni, and 73.1% for Mo. The observed recovery hierarchy is consistent with thermodynamic predictions from Ellingham diagram analysis and is corroborated by a CALPHAD-based simulation in FactSage 8.4 that reproduced the experimental ingot composition to within 2% for all four elements of interest. Total electrical energy consumption was 74 kWh, corresponding to approximately 1.29 kWh per kilogram of recovered material, substantially lower than the approximately 22 kWh/kg required for virgin stainless steel production and the approximately 5 kWh/kg associated with conventional scrap remelting. Economic analysis yielded a net value generation of INR 407.6 (GBP 3.33) per kilogram of grinding dust processed. The output ingot composition closely resembles the input electrode grade, enabling direct reuse as an ESR electrode. A generalised thermodynamically grounded process selection framework is additionally proposed to extend this methodology to other steel grades and specialty alloys.

## Introduction

**Figure 1:**
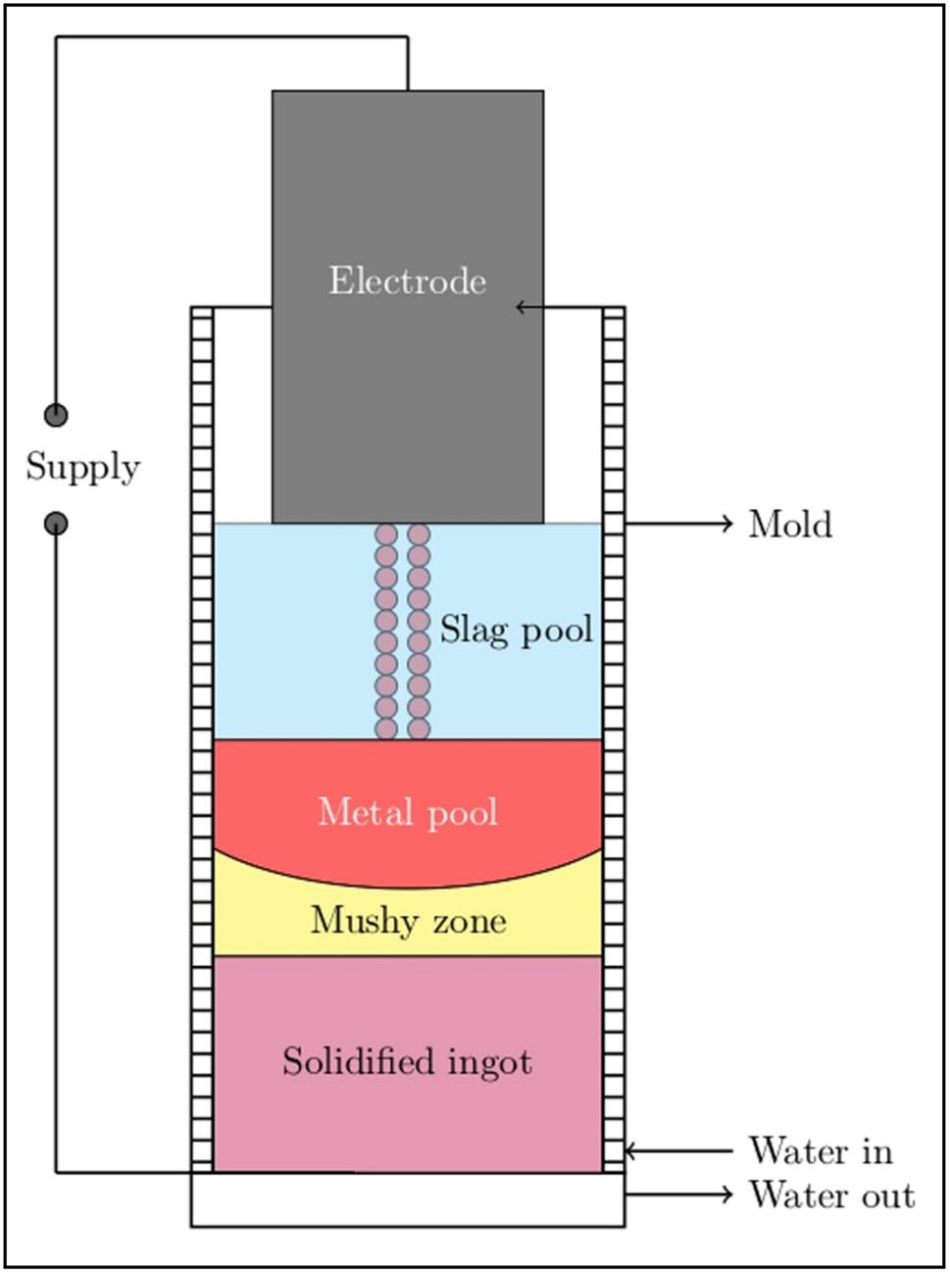
Setup of ESR.

ESR (Electroslag Remelting)^1,2^ is a secondary refining technique employed for the production of high-quality steels having uniform composition with minimal inclusions and micro segregation^3^. Steel produced by ESR is used in a wide range of niche applications, spanning aerospace components, biomedical applications and armor-grade systems, to name a few^4,5^.The process of ESR involves a so called impure steel electrode (which is to be purified), getting dipped into a large cylindrical vessel containing an artificially produced melt pool of slag mainly composed of $CaF_2$, *CaO* and $Al_2O_3$ ^6,7^. The slag typically has poor electrical conductivity and low density compared to steel, hence it forms a distinct layer atop the molten metal. A schematic of the ESR setup is presented in Figure 1. An electric current is applied to the apparatus, with one terminal connected to the electrode and the other to the base of the vessel. This current can be either alternating (AC) or direct (DC)^8,9^. Due to the slag’s low electrical conductivity, Joule heating melts the electrode tip, forming molten droplets that pass through the slag and collect at the bottom while impurities react with the slag, causing de-oxidisation and de-sulphurisation.^10^. Fluxes added to the slag promote impurity removal by forming insoluble compounds. As the less dense slag floats above molten steel, oxidising agents convert impurities such as carbon, silicon, and manganese into oxides that dissolve into the slag, while purified metal droplets settle at the bottom, forming a pool of high-purity metal.^11,12^.

It is observed that the surface of the electroslag remelted ingot, however, is often not perfectly smooth and contains defects like surface cracks and pores (primarily due to faster cooling at the surface than the core and non-uniform Joule heating along the interfaces)^13,14^. Such surface defects are undesirable (also out of specifications provided by customers) in ESR ingots, as cracks act as stress concentrators^15,16^ and may lead to fatigue-induced failure under service loading. Therefore, the ingot surface is typically ground to improve smoothness and reduce the risk of premature failure. Although grinding is used to prevent surface-related failure in remelted ingots, it generates fine dust that poses environmental and occupational health risks ^17,18^. Since ESR is used to produce high-quality steels, the grinding dust contains significant amounts of valuable metallic elements. Its proper management and utilisation are therefore important to reduce health and environmental risks and to enhance steelmaking efficiency through recycling and valorisation, supporting circular economy objectives. ^19^.

Various studies have explored the utilization of conventional steel-making wastes, such as furnace dust^20^, mill scale^21^, and sludge^22^, as alternative burdens in blast furnaces and electric arc furnaces ^23–25^. Because these conventional wastes are largely oxide-rich, they typically require pre-treatment processes such as granulation into binder-assisted, self-reducing briquettes ^26^. These briquettes are then used as supporting charge in existing reduction operations in blast furnaces or EAFs or BFs. However, ESR grinding dust is predominantly metallic with negligible oxide content, allowing direct use as a charge without agglomeration or pre-reduction. It is also chemically purer and more valuable than conventional steelmaking dust, though briquetting is unsuitable due to impurity introduction. With the ongoing shift in the steelmaking industry towards electric arc furnaces (EAFs), the effective utilization of such dusts in EAFs is limited due to the oxidizing furnace atmosphere, which promotes the loss of valuable alloying elements such as chromium and molybdenum^27^. In contrast, induction furnaces, operating under a comparatively neutral atmosphere, offer a more suitable route for the utilization and recovery of metal-rich grinding dusts.

Although various studies have been undertaken in the area of valorization of mill scale and other steel making solid wastes, most of those studies are typically focused on BFs and EAFs. The study on IFs remain highly unexplored. In this study, an induction furnace–based route is proposed for the recovery of iron and other high value metals like chromium, nickel, and molybdenum from ESR grinding dust. The methodology is described in the section 2, and its effectiveness is evaluated through pilot-scale trials conducted at an industrial steel plant. In the discussion section, the economic and environmental feasibility of the proposed method has been discussed.

## Material and methodology

**Figure 2:**
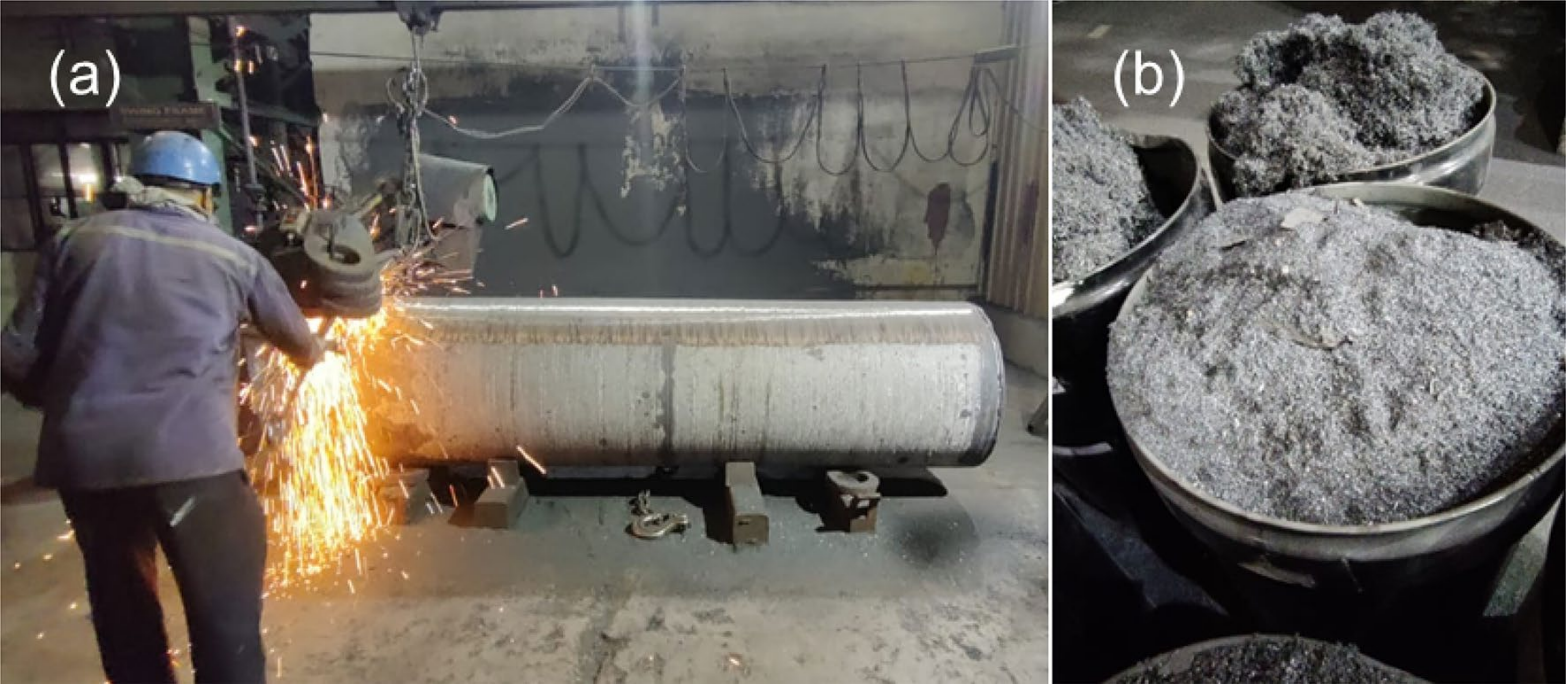
Photos of the methodology - (**a**) Grinding operation, (**b**) Collected grinding dust.

**Figure 3:**
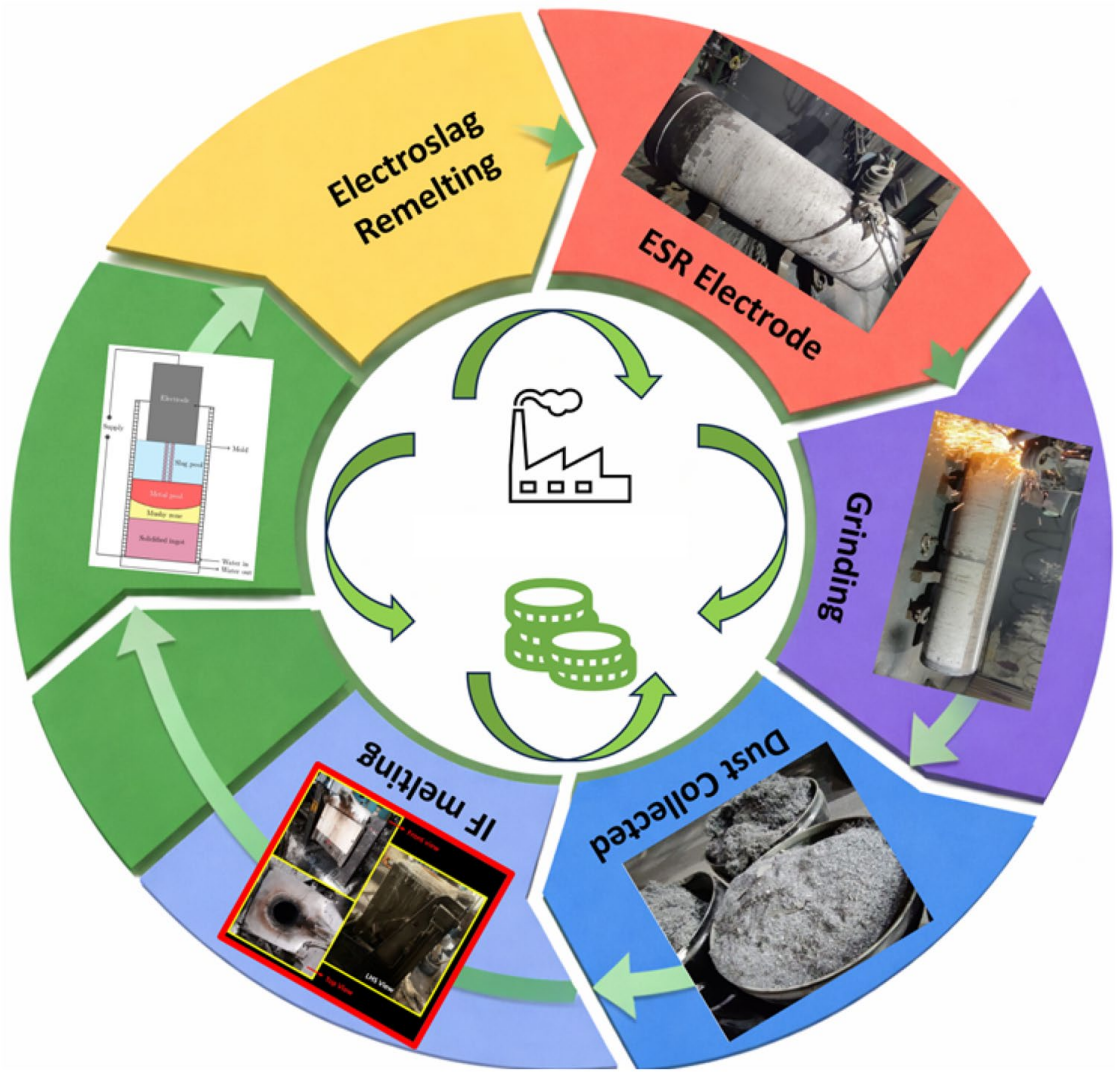
Proposed method for recovery of metals from ESR grinding dust.

In this work, grinding dust was collected from the surface grinding of ESR ingots of SS321 grade as shown in Figure 2 (a) and stored (Figure 2 (b)). After collection, the dust was dried for two hours and stored under controlled conditions. The processed dust consists of fine particles, which makes direct melting challenging. To recover metals from the dust, an induction furnace was selected as a suitable route, as it enables melting of conducting materials through eddy-current-induced Joule heating ^28^. However, the small particle size limits the magnitude of eddy currents that can be induced directly in the dust. In addition, the grinding dust exhibits low electrical conductivity and contains a significant fraction of non-magnetic phases. To address this, a liquid metal pool of the original electrode material was first created to provide electrical conductivity and act as a thermal reservoir ^21,29^. Magnesia ramming mass based inner lining was used for the induction furnace. Once the electrode material had melted, the grinding dust was gradually added to the melt. The possibility of applying a pre-treatment step to the grinding dust was considered, however it was deemed unnecessary due to the characteristics of the dust. The reasons for the same have been explained in the results section. Heat transfer from the molten metal to the dust particles, primarily through convection and radiation, led to their melting. The resulting melt was then solidified and recovered, either for reuse as an ESR electrode or for other suitable applications. The overall process route, from ESR electrode production to dust recycling, is schematically illustrated in Figure 3.

**Figure 4:**
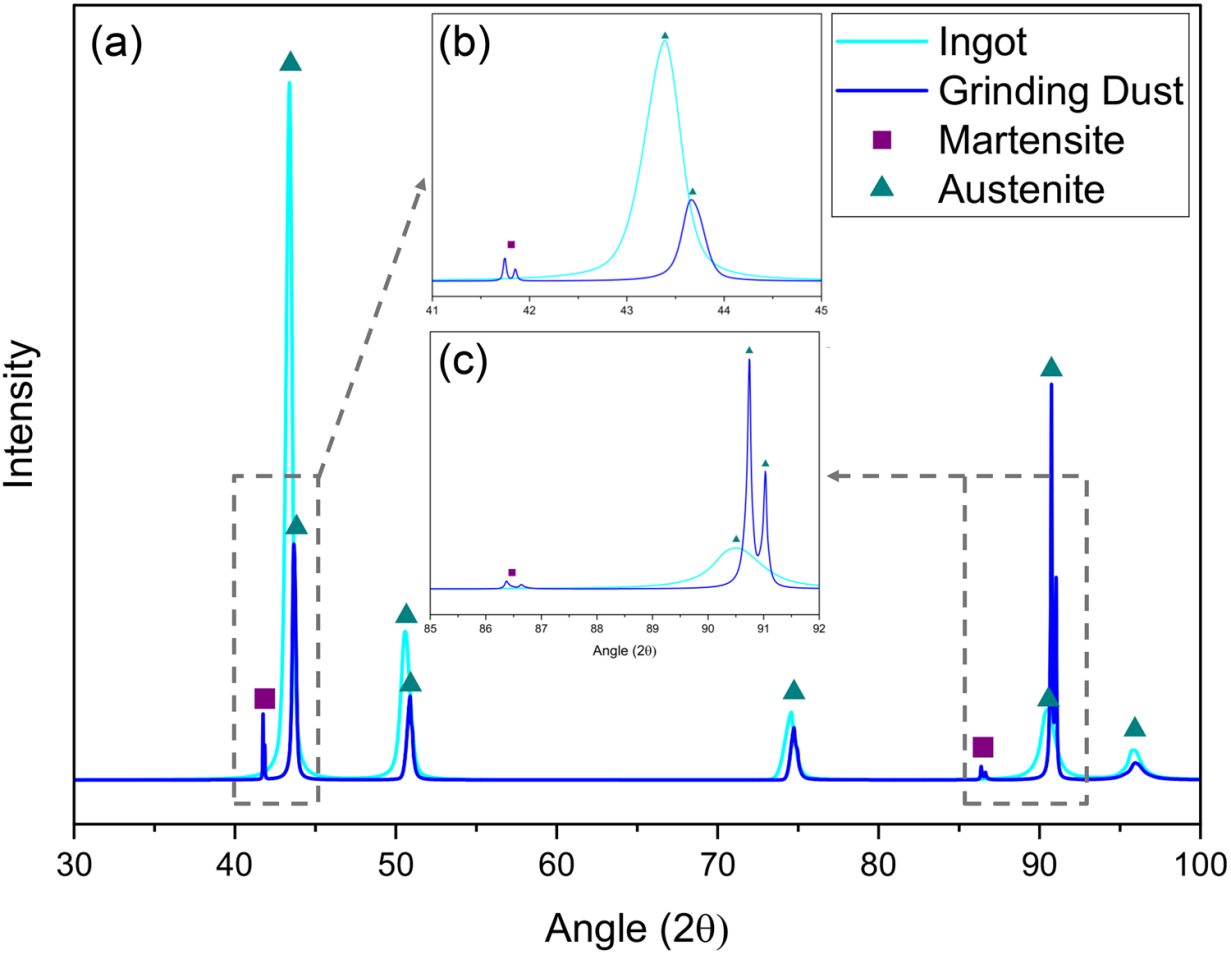
XRD of the ESR treated ingot and grinding dust.

**Figure 5:**
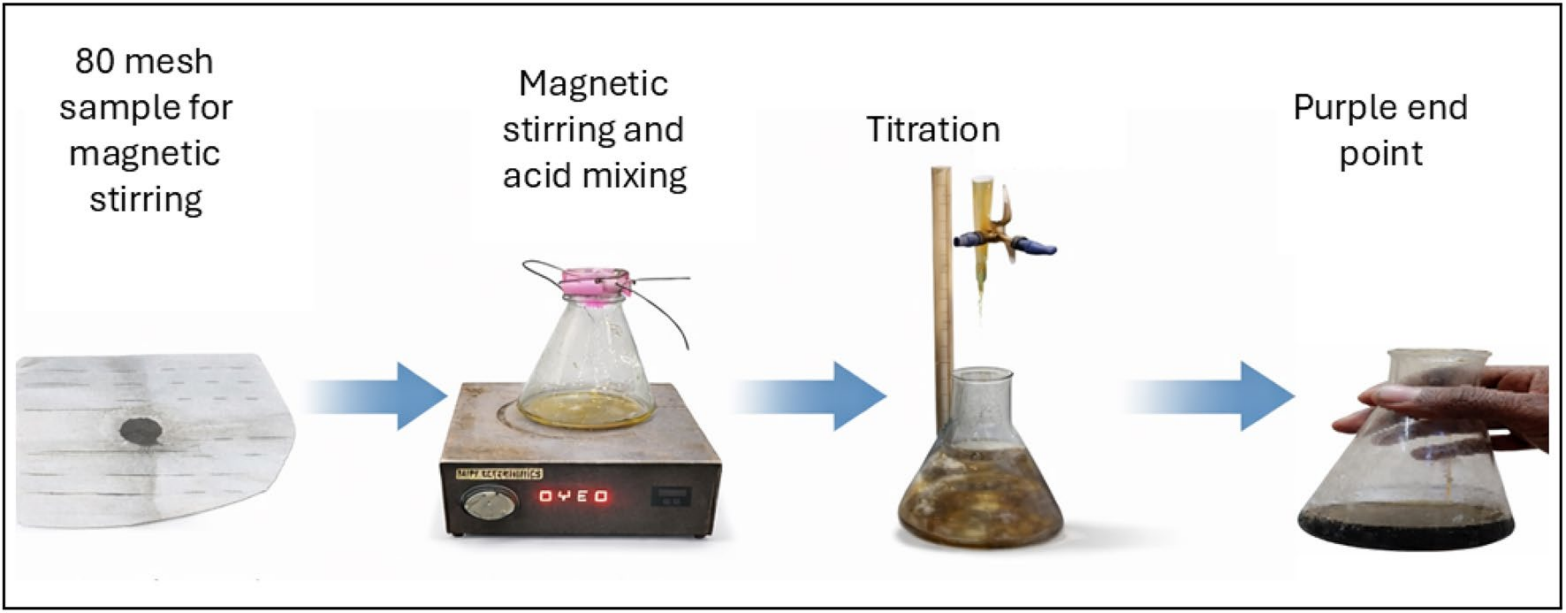
Experimental procedure for the wet analysis of grinding dust.

## Results and discussions

**Figure 6:**
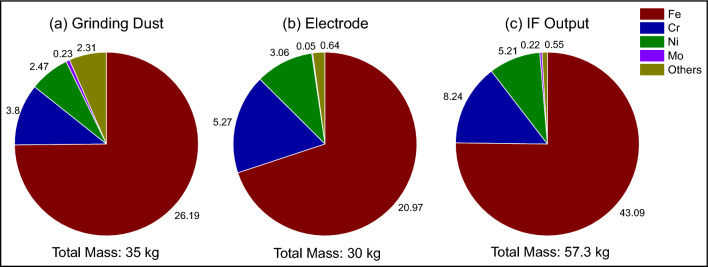
Composition by mass (**a**) Grinding Dust, (**b**) Electrode and (**c**) Induction Furnace output. All values are in kilograms.

### Characterization of dust

Once the dust was collected and dried in a controlled environment, its chemical composition was evaluated using X-ray Fluorescence (XRF) performed on a Thermo Scientific ARL 3460 metals analyser; which is given in table 1

**Table 1:** Chemistry of grinding dust powder and electrode.

Component	Fe	C	Mn	Si	S	P	Cr	Ni	Mo	Al	Ti	Cu	V
ESR Dust (Wt .%)	74.83	0.12	1.94	1.65	0.18	0.1	10.87	7.05	0.65	0.81	0.19	0.57	0.11
Electrode (Wt .%)	69.91	0.06	1.03	0.51	0.1	0.02	17.56	10.21	0.18	0.1	0.05	0.14	0.12

The dust is rich in iron and other value elements like chromium, nickel and molybdenum. To evaluate the nature of iron present in dust, wet analysis as depicted 5, was carried out to quantitatively determine the amount of metallic iron in the sample. A 100 mg sample of grinding dust was placed in an air-sealed conical flask with 15 ml water and 35 ml Ferric Chloride solution. The solution was then stirred for 45 minutes and treated with 0.1 N $K_{2}Cr_{2}O_{7}$ until a purple endpoint confirmed metallic iron. The ferrous content (M.F.) was calculated using Equation 1.

1 $$\begin{aligned} \text {Metallic Fe} = \frac{V_t * 0.005585 *100}{W * 3} \end{aligned}$$

Where $V_t$ is amount of $K_2Cr_2O_7$ in ml consumed during titration and W is the Weight of sample in gm.The ferrous content value obtained from the wet analysis was 75.61%. This is in close agreement with the percentage results of chemical composition obtained using XRF as given in table 1. It can be further seen from table 1 that, the dust is rich in high value metals like chromium, nickel and molybdenum whose recovery is the main purpose of this study.

To get a better idea of the phases present in the grinding dust, XRD phase analysis was also performed on a Rikagu Smartlab X-Ray Diffractometer. The raw data was smoothened out and analyzed with the help of a commercial software; the COD database was used to check the peak identifiers which is depicted in Figure 4. To capture the effects of grinding process on the phases present in ESR dust, the XRD of a small sample of the ESR ingot was also performed as a baseline. It can be seen from Figure 4 that the ingot predominantly consists of the austenite phase. The peaks associated with this austenite phase include the FCC phase for iron (COD ID: 96-900-8470) and nickel (COD ID: 96-901-3032). It should be noted that this phase is one single phase consisting of an iron matrix with all other elements dissolved in the form of a solid solution.The obtained results are consistent with already reported data for the XRD of this steel^30^. The pattern corresponding to the grinding dust, however, also contains Martensitic peaks likely a result of the mechanical working process occuring during the grinding operation, causing a TRIP effect in the steel^31^. The location of these peaks are consistent with the findings of other contemporary works^32^. The grinding process also results in the change of relative intensity of the Austenitic peaks. The Austenitic peaks are also slightly shifted with respect to the ones of the pure ingot; this is consistent with expected lattice straining caused by martensite formation^33^ and the mechanical working process^34^. The peaks of both, ingot and grinding dust are relatively wide, likely due to the various solute atoms present inside the Austenitic matrix, resulting in a large variation of the lattice parameter. Also, there is no evidence of oxidation in the grinding dust. Generally, there are multiple peaks for angles less than $40^{\circ }$, which correspond to the various oxides and other compounds formed during oxidation process as shown in an earlier study^21^. It should be noted that the two peaks arising at 91 degrees of the grinding dust are a result of the characteristic $K_\alpha$ radiation from the copper anode, which consists of two slightly different wavelengths. This is a well documented phenomenon known as a ’doublet’ peak and both of the peaks correspond to the same plane of the Austenite phase^35^. Similarly, the double peaks obtained for the Martensite phases are also a well documented phenomenon^36^. Grinding dust characterization was carried out to assess the need for pre-treatment such as pre-reduction. Pre-reduction is generally required when the powder contains significant oxide content. However, the dust in this study originates from electro-slag remelted (ESR) ingots and therefore exhibits high purity with negligible oxides and sulphides, as confirmed by XRD analysis. Consequently, the particles could be directly integrated into the melt pool, as discussed.

To establish the melt pool, 30 kg of electrodes with the composition listed in Table 1 were used. The electrodes correspond to the same grade as the electroslag remelted ingot. Melting was carried out in an induction furnace with an initial energy consumption of 42 kWh. Once the melt pool reached 1550 $\vphantom{0}^\circ$C, 35 kg of grinding dust was gradually added to the molten metal, where it melted using the thermal energy available in the melt pool. A thin slag layer formed on the surface and was periodically skimmed before the addition of each subsequent batch of dust. The total electrical energy consumption for the process was 74 kWh. The operating temperature was selected by balancing melt fluidity and oxidation losses. Higher temperatures reduce melt viscosity and enhance electromagnetic stirring in the induction furnace, promoting homogenization and facilitating the transport of inclusions to the slag phase^37–39^. However, excessive temperatures increase oxidation of alloying elements. The melting point of SS321 is approximately 1400 $\vphantom{0}^\circ$C^40,41^, and a superheat of 100–150 $\vphantom{0}^\circ$C is commonly applied to ensure sufficient fluidity^42^. Studies on stainless steels indicate that oxidation of alloying elements becomes significant above  1550 $\vphantom{0}^\circ$C^43^. Accordingly, 1550 $\vphantom{0}^\circ$C was selected as the processing temperature. A lollipop sample was extracted to determine the composition of the resulting ingot, summarized in Table 2. Subsequent analysis was performed to evaluate the recovery efficiency as discussed.

**Table 2:** Chemistry of obtained output ingot from IF.

Elements	Fe	C	Mn	Si	S	P	Cr	Ni	Mo	Al	Ti	Cu	V
(Wt .%)	75.19	0.13	0.34	0.17	0.05	0.03	14.38	9.09	0.38	0.01	0.001	0.14	0.10

### Mass Balance and efficiency calculations

 Figure 6 (a) and (b) show the quantitative presence of elements in the charge mix. 30 kilograms of electrode material was used and 35 kilograms of grinding dust was used. The final weight of the ingot obtained from the induction furnace was 57.3 kilograms, details of which can be seen in Figure 6 (c). The remaining mass loss is primarily attributed to slag (7.7 kg), interaction with the furnace lining, and minor handling losses during charging^44^. In addition, partial oxidation and transfer of alloying and impurity elements to the slag phase are expected during induction melting, contributing to the observed material loss ^21^.

**Figure 7:**
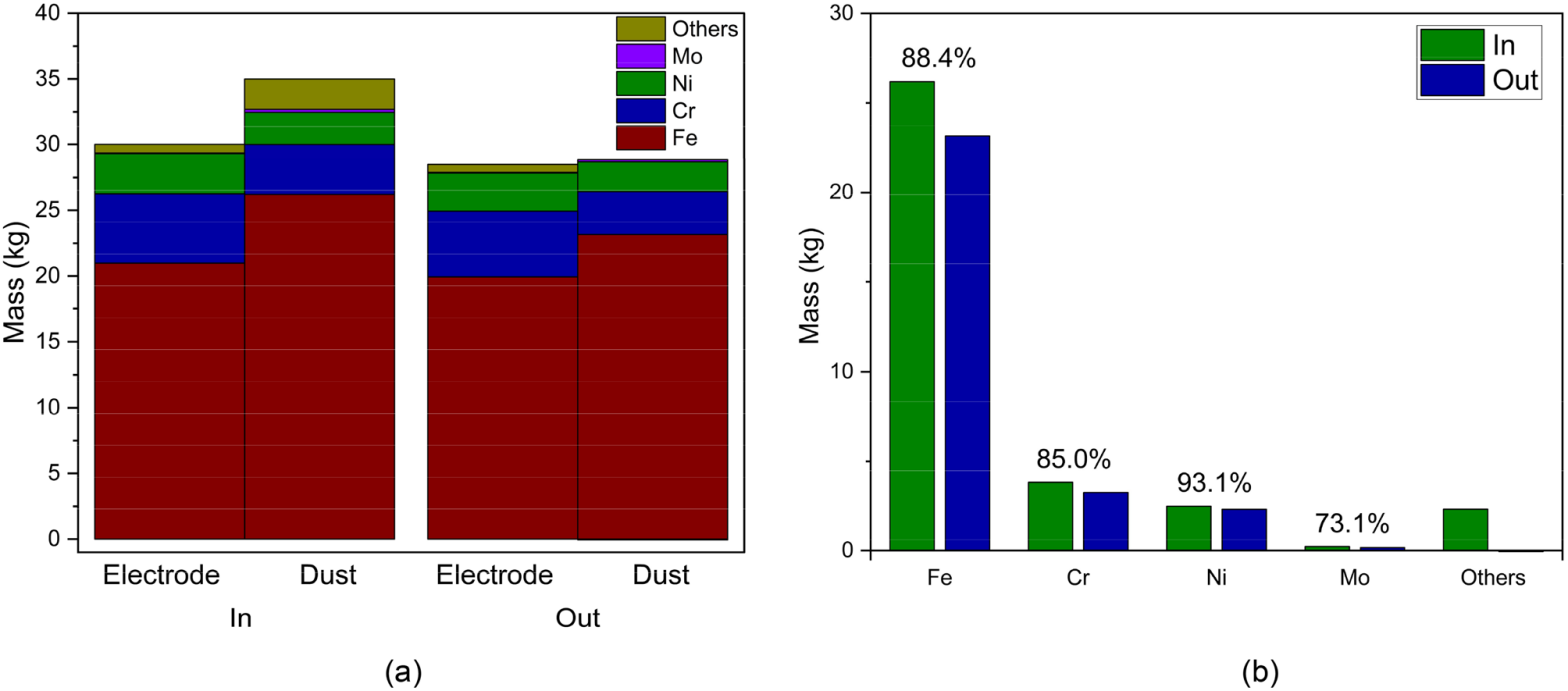
Comparison of input and output masses for each element. (**a**) Elemental composition and mass balance; (**b**) Recovery efficiencies of the elements.

A mass balance for Fe, Cr, Ni, and Mo was carried out to assess the efficiency of the process. The input mass of each element was calculated from its mass fraction in the feed materials and their respective total masses, accounting for contributions from both the electrode material and the grinding dust. The resulting mass balances are presented in Figure 7 (a), with the corresponding raw data listed in Table 3.

The elemental composition of the output ingot reflects contributions from both the initial melt pool (electrode material) and the added grinding dust. Quantifying the fraction originating from the grinding dust is therefore essential for evaluating process efficiency. In this work, a conservative assumption was adopted in which 95% of the metals originating from the melt pool material were recovered, with the remaining contribution attributed to the grinding dust. This assumption is based on long-term average recovery data from induction furnace operations at Saarloha Advanced Materials Private Limited and is consistent with reported literature values ^45^. Under this assumption, the recovery from the grinding dust was determined using Eq. 2.

2 $$\begin{aligned} \eta _{i} =\frac{W_{output} - 0.95 * {W_{input,Electrode}}}{W_{input, GD} } \end{aligned}$$

Here, the quantities $W_{output}$, $W_{input,Electrode}$ and $W_{input, GD}$ are the total output weight, weight from the electrode and weight from the grinding dust respectively. By applying this formula, the final weights of the elements and the recovery have been determined. The total input and output quantities of the various elements in the grinding dust can be seen in Figure 7 (b). The efficiency was observed to be 88.4% for iron, 85% for chromium, 93.1% for nickel and 73.1% for molybdenum.

**Table 3:** Qualitative analysis of mass of different elements in the input and output material of IF trials.

Item	Fe	Cr	Ni	Mo
Electrode (kg)	20.97	5.27	3.06	0.05
Grinding Dust (kg)	26.19	3.8	2.47	0.23
Output from Electrode (kg)	19.92	5	2.91	0.05
Output from Grinding dust (kg)	23.16	3.24	2.3	0.17
Process Efficiency (%)	88.43	85.03	93.1	73.16

From the above analysis, it is apparent that the proposed process has favorable economic and energy implications. As ESR is used for producing high-quality steels for specialized applications, the associated grinding dust is chemically pure and rich in valuable alloying elements, making it suitable for metal recovery. Although a small loss of the original electrode material is observed (approximately 5%), more than 80% of the metals present in the grinding dust are recovered, supporting circular economy objectives.

**Figure 8:**
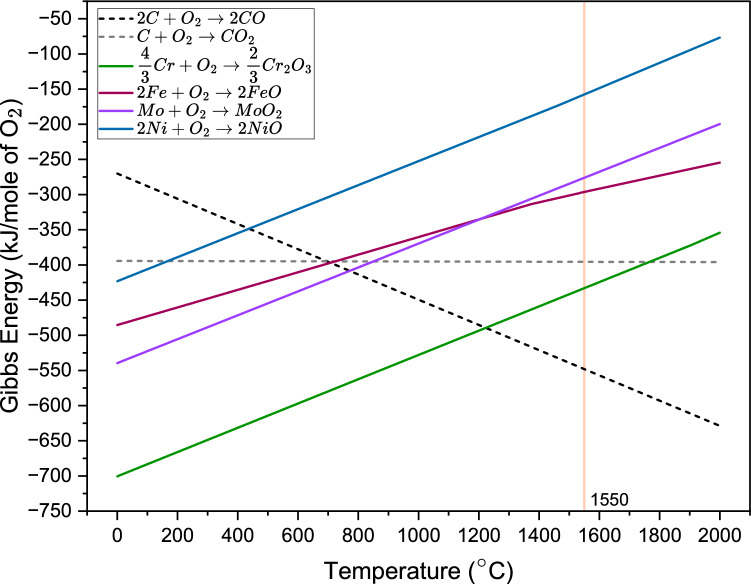
Ellingham diagram for the metals and carbon.

### Thermodynamic analysis of the obtained results

In this section, we attempt to explain the results and their mechanisms with the help of a general first-principles thermodynamic framework. The hypothesis is validated with the help of a CALPHAD model developed in the commercial software FactSage 8.4. With regard to the non metallic elements present in the initial melt pool composition, a considerable amount of desulphurization has taken place during the process. The sulphur concentration dropped to 0.05% compared to 0.18% and 0.1% in the ESR dust and electrode material, respectively. The observed effect can be explained by an earlier work conducted by Motlagh ^46^. According to his analysis, a slight oxidative atmosphere, due to oxygen in the air, may cause desulphurization to a large extent due to the formation of oxides of sulphur. Additionally, moisture in the air may adsorb onto the surface of the induction furnace, subsequently dissociating into atomic adsorbed oxygen and hydrogen atoms. These atoms react with the sulphur present in the melt pool to form hydrogen sulphide. It is also observed that the concentration of silicon has reduced in the output metal compared to the input materials. This is a result of direct oxidation of the silicon present in the material, as shown by Dotsch ^47^. This is further supported by the formation of a slag layer observed during the melting process. Additionally, the phosphorus content in the output ingot is lower than that of the input materials, implying that dephosphorization has occurred. This is again a result of phosphorus oxidation, as shown by Bedarkar and Singh^48^.

The extent of oxidation reactions occurring in the induction furnace melt is predominantly explained by the thermodynamic properties of the reactions under consideration. The Ellingham diagram^49^ is a convenient method to compare the standard Gibbs free energy change of various oxidation reactions as a function of their temperature. The Ellingham diagram for the metals of interest in the present study has been presented, along with the reactions of carbon for reference, in Figure 8. In general, the lower the Ellingham curve of a material, the greater the proclivity of oxidation. Hence, a material oxide whose Ellingham curve lies atop a second material will be reduced to metal by the latter element, which will get oxidized. It is visible that the Ellingham curve for nickel is above every other major constituent of the steel at 1550 $\vphantom{0}^\circ$C. Hence, whenever nickel oxide is formed inside the melt, atoms of iron, molybdenum or chromium have a tendency to reduce it back to nickel, while simultaneously getting oxidized. This explains the highest recovery rates of nickel amongst the four main components of the steel.

Another important aspect to consider during the analysis of metallic elements is to account for side reactions, such as the conversion of oxide to a salt due to reaction with a chemical species present in the slag. The Ellingham diagram merely shows the tendency of materials to react with oxygen, however, the elements may be exposed to other reactants such as silicon, sulphur and other inclusions of the metals; or constituents of the atmosphere such as carbon dioxide; or other foreign materials such as dust in the air, the inner lining of the furnace, etc. The case of molybdenum is of particular interest in the present steel grade. It can be observed that the recovery rates for iron, chromium and nickel are all above 85%, while the recovery rate of molybdenum is 73.1%, which is considerably lower. This can be attributed to the fact that molybdenum trioxide ($MoO_3$) is strongly acidic. When $MoO_2$ is formed in the melt, it may convert to $MoO_3$, which is acidic in nature. This acid reacts rapidly with the materials of the flux or the inner lining of the IF, which are basic in nature. Additionally, $MoO_3$ is highly volatile at steelmaking temperatures and is prone to sublimation^50^. This lowers the molybdenum oxide concentration below its equilibrium value, driving greater conversion from metal to oxide in accordance with Le Chatelier’s principle and thereby reducing process efficiency. In contrast, the oxides of nickel, iron, and chromium are either basic or amphoteric^51^, resulting in higher efficiency. Consequently, the unrecoverable fractions of these elements are physically lost to two primary streams: the basic slag (as complex oxides) and the exhaust gases (as volatilized compounds).

**Figure 9:**
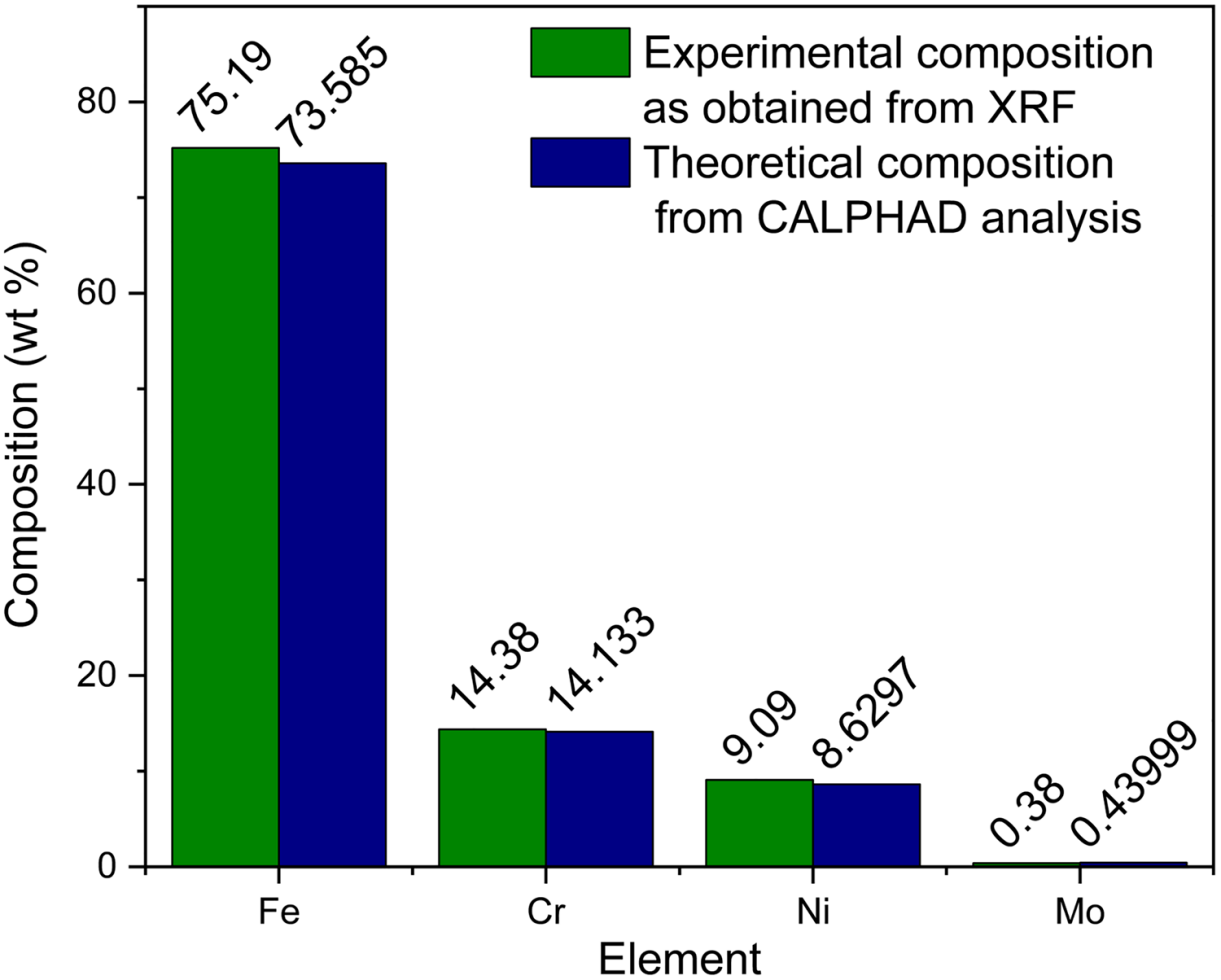
Comparison of experimentally determined composition of output ingot with theoretically determined composition.

The above discussion qualitatively describes the cause of the obtained results. To obtain a quantitative description based on thermodynamics and for the validation of the hypotheses discussed above, a CALPHAD based model was adopted on the commercial software FactSage 8.4^52^. The input was set to be the composition of the 65 kg ingot formed by the mixing of the electrode and grinding dust. For the sake of simplicity, the inner lining of the induction furnace was assumed to be composed entirely of *MgO*. Sufficient oxygen was introduced into the simulation to facilitate slag formation. It was determined that an effective addition of 7.7 g O$\vphantom{0}_2$/kg of metallic charge accurately replicated the experimental ingot composition. This lean oxidation level is attributed to the formation of the slag barrier. Isothermal condition was applied on the final output. The resulting output ingot had a composition very close to the experimental observations. The comparison of the composition obtained (provided in table 2) and the composition theoretically predicted with the help of FactSage have been presented in Figure 9. The composition of all four elements of interest - Fe, Cr, Ni and Mo in the output ingot is within 2% of the experimentally obtained composition. As expected, the slag is composed of various oxides, silicates and sulphides. The mass of the slag, however, is approximately 2 kilograms in the simulation, which is less than the loss in weight of 7.7 kilograms. This extra loss of $\sim 5.7$ is attributed the loss of metal during skimming out of the slag, apart from gaseous and handling loss. Some amount of molten metal stuck to the slag due to adhesive interactions or entrained in the slag is lost during skimming process, causing this loss. The quantitative amount of loss is about 9%, a figure consistent with the literature values of 10%^53^.

### Economic, energetic and environmental implications

The process exhibits high energy efficiency, as the only significant energy input is that required for operation of the induction furnace. It was observed that retention of the slag layer on the molten bath reduces radiative heat losses to the surroundings, thereby contributing to lower overall energy consumption.^54^. The total energy consumption for the recovery process was 74kWh. The energy required per kilogram of recovered material is thus 1.29 kWh. This value is considerably lower than the energy required to produce the same amount of alloy from the ore. A report by Johnson et. al.^55^ suggests that the energy requirement for the production of virgin stainless steel through the extractive metallurgy route requires approximately 22 kWh of energy per kilogram. The study also finds that current cost of stainless steel production through the remelting of scrap requires approximately 5 kWh of energy per kilogram. Hence, it can be concluded that the proposed method is energetically favorable as the energy required is considerably lower than the energy required to extractively obtain the same amount of metal.

Table 4 summarizes the input costs and the corresponding value of the recovered alloying elements for the IF-based trial. The unit prices assigned to Fe, Cr, Ni, and Mo correspond to prevailing market rates at the time of the study. The total operational cost, including raw materials, electricity, manpower, and miscellaneous expenses, is Rs. 10,356.5, while the cumulative market value of the recovered metals (Fe, Cr, Ni, and Mo) amounts to Rs. 24,622.7 (Table 4). The difference between these values represents a net value generation of Rs. 14,266.2 for the batch processed. When normalised with respect to the quantity of ESR grinding dust used (35 kg), this corresponds to approximately Rs. 407.6 (GBP 3.33) of net value generated per kilogram of dust, despite the dust itself having a nominal market cost of Rs. 5 kg$\vphantom{0}^{-1}$. Expressed differently, the net value generated is approximately $81\times$ the cost of the dust feedstock. It is noted that the present analysis is based on batch operation and includes the full electrode consumption required to initiate melting. Operation under a continuous tapping, with retention of a molten heel and successive dust charging, would reduce consumable requirements per cycle and thereby increase the net value realized per unit mass of dust processed. During an industrial scale operation, the melt pool will be created initially with the help of the electrode only once and regular tapping will ensure a continuous operation; thus greatly reducing the input costs. Additionally, the slag layer which is separated in the induction furnace can be reused in other industries such as agriculture^56–58^, thus promoting a circular economy and further adding to the value of the process.

**Table 4:** Cost analysis of IF-based trial.

Input Cost			Output Value		
Material	Quantity	Cost (Rs.)	Material	Quantity (kg)	Cost (Rs.)
ESR dust (Rs. 5/kg)	35 kg	175	Fe (Rs. 30/kg)	43.09	1292.7
Electrode (Rs. 250/kg)	30 kg	7500	Cr (Rs. 1500/kg)	8.24	12360
Electricity (Rs. 10/kWh)	74 kWh	740	Ni (Rs. 2000/kg)	5.21	10420
Manpower (Rs. 2000/day)	–	1000	Mo (Rs. 2500/kg)	0.22	550
**Subtotal**		**9415**			
Miscellaneous (@10%)		941.5			
**Total input cost**		**10,356.5**	**Output value**		**24,622.7**

It should be noted that the proposed method, while being energetically and economically efficient, also exhibits high recovery efficiency as compared to the results of other valorization studies. While comparison of recovery efficiency is not completely relevant when the waste is considered an alternative burden in BF/EAF as the ’grade of steel’ itself is lost and becomes part of the BF/EAF output, efficiencies reported with the leaching route can be compared. Petranikova et. al.^59^ have developed a method for the valorization of steelmaking dust through a leaching based approach. In their study, they report a recovery efficiency of $\sim$60 % for molybdenum for a sample having low initial molybdenum concentration of 0.61% and $\sim$70% recovery efficiency for a sample having high initial Mo content of 2.65%. Meanwhile, in the present study, we report an efficiency of 73.1%. Rudnik et. al.^60^ pre-treated the grinding dust by grinding in a ball mill to increase the leaching efficiency; and followed it up with a leaching step for one hour. They have reported a maximum of $\sim$ 50% recovery of iron from their approach. This is consistent with the findings of another study, who reported 48% recovery^61^. In the present study, 88.4% of the iron was recovered.

It is crucial to consider the environmental implications of the proposed process. Steelmaking waste is generally classified as hazardous industrial waste due to the presence of dangerous metals such as Mo, Ni, etc. If these leach into the groundwater and the ecosystem, they cause significant harm to the plants and animals of the local biosphere^62^. Grinding dust, in particular, is particularly more dangerous as the small dust particles also contribute to air pollution. Its single step recycling with the proposed methodology is greatly beneficial for the environment if implemented properly. However, care should be taken that the vapors arising during the melting process are not released into the atmosphere. Studies performed on animals have shown that inhaling gases such as $MoO_3$ vapors or their dispersed nanoparticles cause cellular damage and chronic exposure is harmful^63^. For large scale implementation, it is hence important that a duct be installed to capture these harmful gases. Additionally, the hazardous slag components should be clearly classified and treated before disposal to ensure that environment guidelines are not violated^58^. This is essential because most of the lost elements that have not sublimated in any form end up in this slag.

**Figure 10:**
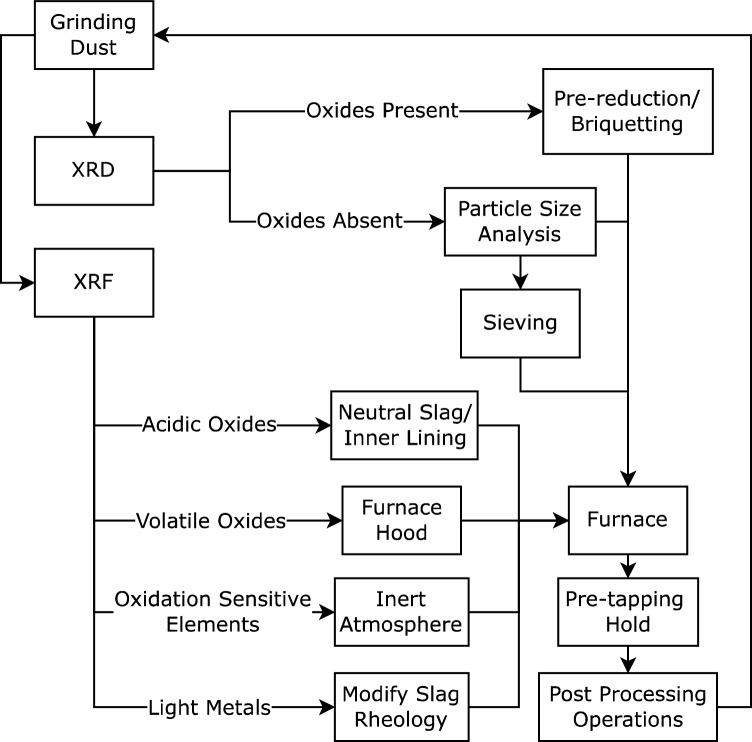
Determination of process parameters for other alloys.

### Extension of concept to other materials and larger scales

The novel method of grinding dust valorization presented in the present work has been successfully applied in the pilot project presented for the SS321 stainless steel grade. However, for the method to have significant scientific implications for the steelmaking industry and for it to contribute meaningfully towards the steel recycling targets set by the governments, it must be versatile and scalable. In this section, we attempt to use basic principles of chemistry and thermodynamics already discussed above to extend the proposed method to other grades of steel. The main aim is to design a process flow which ensures good recovery rates for each material. Additionally, we use already existing literature to determine the impacts and effects of large scale implementation and the optimization of process parameters at larger scales. The determination of process parameters to optimize for recovery for any metallic material can be observed in Figure 10. In the subsequent text, each of the underlying concepts have been briefly discussed.

The first step towards understanding the required processing is to thoroughly understand the physical and chemical characteristics of the grinding dust. As presented in the present study, it is most important to determine the chemical composition of the dust; this can be done with the help of XRF or other spectroscopy techniques. The effect of the composition on the parameters is discussed in more detail later. In addition to the composition, it is useful to determine the phases present in the dust with the help of XRD. If there are significant oxide phases present in the dust, it will be of benefit to perform a pre-reduction step. In addition, a particle size analysis can be performed to check for the presence of too large particles, which can be sieved out. These steps generally will not be required for ESR grinding dusts due to their purity, but should be done nonetheless as a confirmatory procedure.

The next step is to identify the processing conditions required for the process based off the chemistry of the composition of the grinding dust. If a significant portion of the dust is composed of elements which are prone to form acidic oxides, basic components in the slag and inner lining of the induction furnace must be avoided. A general principle is that the metal oxides in which the metals exhibit a high oxidation state (+5 - +8), the oxide will be acidic^64^. These metals include V, Nb, Ta, Cr, Mo, W and Mn; and their corresponding acidic oxides are $V_2O_5$, $Nb_2O_5$, $Ta_2O_5$, $CrO_3$, $MoO_3$, $WO_3$ and $Mn_2O_7$ respectively. While some of these metals do exhibit alternative oxides in which they exhibit lower oxidation states and are not acidic, there is a possibility of the formation of these acidic oxides if the processing temperatures thermodynamically favour their formation. Obviously, the processing temperature is a function of the melting point of the metal rather than the stable oxidation state of such metals. Hence, if there is a possibility of the formation of these oxides, basic inner linings such as magnesia should be avoided. Neutral linings such as zirconia should be used instead^65^.

There are certain metallic oxides whose boiling point may lie below the process temperature. These ’volatile’ metal oxides may evaporate. Such oxides must be identified on the basis of the operation temperature of the furnace. For the temperatures involved during steelmaking, certain oxides of Mo, W, Cr, Sb, As and Pb are volatile. Their oxides: $MoO_3$, $WO_3$, $CrO_3$, $Sb_2O3$, $As_2O_3$ and *PbO* all have their boiling point below 1500 $\vphantom{0}^\circ$C^66^. Fundamentally, evaporation occurs when the partial pressure of the component is less than its vapour pressure. Hence, the evaporation process can be controlled by maintaining the partial pressure close to the vapour pressure. This can be achieved with the help of a furnace hood over the induction furnace. This furnace hood will not allow the already vaporized gas to escape, and when its concentration is equal to the vapour pressure, no further evaporation will occur.

Metals such as Ca, Mg or Al, whose oxidization curve lies low in the Ellingham diagram, are highly prone to oxidization. While they were not present in the material used for the pilot project, they are highly susceptible to oxidation in grades having a significant portion of these metals. If these elements are to be preserved, it is highly recommended that an inert gas atmosphere be used in addition to a furnace hood. This can be achieved by using an inert gas air jet inside the furnace hood.

Light metals such as Li, Mg and Al are susceptible to a phenomenon known as entrainment during induction furnace steelmaking. The entrainment phenomena of metal in the slag layer have been extensively studied using water models^67^ and X-ray transmission photography^68^. It has been observed that gas bubbles transport the metal to the melt-slag interface, and the bursting of those bubbles at the slag interface results in the entrapment of the metal in the slag. The settling of the trapped metal droplet is arduous due to the small size and the inherent high viscosity of the slag layer. The entrainment of metal into slag reduces process efficiency. To effectively mitigate mutual entrainment between the metal and slag phases, operations must optimise slag rheology. While maintaining a high slag viscosity is explicitly desirable because it makes inclusion re-entrainment more difficult^69^, the slag must not become overly saturated with solid phases. The presence of solid particles in the liquid slag can attach to entrained metallic droplets, creating a combined entity with a lower apparent density; this acts as a mechanical trap that severely hinders the ability of the metal droplets to settle back into the liquid bath. The quantitative steps involved in the optimization of slag rheology to prevent entrainment are beyond the scope of this study; however, the references provided present guidelines for the implementation in the presence of light elements such as the ones stated above, as they are more susceptible to entrainment.

Entrainment can also occur the other way round: small slag particles can be entrained inside the metal phase^69^. This would cause a degradation of the quality of the output ingot by introducing inclusions at the top surface. One of the easier approaches to mitigate this involves implementing a pre-tapping “settling period” by reducing induction power. This allows the electromagnetic stirring to subside, thereby reducing the overall flow intensity, resulting in the slag bubbles rising to the top^70^.

The obtained output ingot is also of high purity, due to the pure charge and the slag formation process. However, the final composition is not exactly equivalent to either the input material or the ESR electrode material due to the oxidation losses occuring during the process. This may be rectified by the addition of suitable ferroalloys^71^. These ferroalloys can be added in the induction furnace itself. This allows for accurate composition control, with low cost. Regarding the defects in the output ingot, the pre-tapping hold is crucial for the output ingot to be devoid of inclusions and voids. The output ingot can be subsequently subjected to a secondary refining process such as ESR. If there are dust particles or other impurities on the surface of the ingot, a sandblasting step may be required to remove these before the ESR step.

The novelty of the method lies in the fact that the obtained output is of similar composition as compared to the input grade; and the entire process is a single step process. In the case of leaching based approaches, the first step involves leaching the metal into a solution; another step is required to precipitate the metal from the solution. In the case of briquetting and pre-reduction based approaches, the input materials are added as alternate burdens in BFs and EAFs, resulting in them getting mixed with large quantities of steel scrap and iron oxide, which are used as input materials in these furnaces. Hence, the output grade resembles pig iron in the case of BF and the scrap material in the case of EAF , causing a large value loss of the grinding dust in comparison. In the present work, however, the specialty steel grade can be isolated and is just mixed with the initial electrode used to create the melt pool. This melt pool is also of the same grade of material implying that the output material closely resembles the input grade. Also, the batch size of an induction furnace can be altered as per requirement. This flexibility is not available with BFs or EAFs, as they require large batch sizes to operate. The proposed method is applicable not only to SS321 but also to other steel grades, as discussed in the framework above.

## Conclusion

In conclusion, an induction furnace-based route was successfully demonstrated for the recovery of iron, chromium, nickel, and molybdenum from grinding dust generated during surface finishing of electroslag remelted SS321 stainless steel ingots. The proposed method involves the creation of a melt pool in the induction furnace with the ESR electrode material to serve as a heat source for the direct melting of grinding dust, which is subsequently added to the melt pool without any granulation or briquetting. A pilot-scale trial was conducted with the SS321 grade using a 65 kg charge comprising 30 kg of electrode material and 35 kg of grinding dust.

Recovery efficiencies of 88.4%, 85.0%, 93.1%, and 73.1% were obtained for iron, chromium, nickel, and molybdenum, respectively. The observed recovery hierarchy is consistent with thermodynamic predictions, as shown using first principles and proven with a CALPHAD model. The comparatively lower recovery of molybdenum is attributed to the acidic nature and high volatility of MoO$\vphantom{0}_3$ at steelmaking temperatures, which promotes losses to both the slag phase and the furnace atmosphere. The recovery efficiency of molybdenum was, however, still greater than the reported values of 60-70% for leaching-based recycling approaches. The total electrical energy consumption was 74 kWh, corresponding to approximately 1.29 kWh/kg of recovered material, which is significantly lower than the 22 kWh/kg associated with virgin stainless steel and the 5 kWh/kg associated with remelting scrap. Economic analysis yielded a net value generation of INR 407.6 (GBP 3.33) per kilogram of grinding dust processed, with further improvements anticipated under continuous industrial operation.

The output ingot composition was found to closely resemble that of the input electrode material, rendering the recovered product directly suitable for reuse as an ESR electrode following appropriate compositional adjustment, thereby enabling a closed-loop recycling strategy within specialty steel production. A generalised process selection framework was proposed, providing a thermodynamically grounded basis for extending the methodology to other steel grades and specialty alloys. The proposed method offers a single-step alternative to conventional valorization routes such as leaching and briquette-based pre-reduction, while preserving the compositional integrity of the input steel grade and supporting circular economy objectives within the specialty steelmaking industry.

Further studies and industry implementation of this work directly contribute to the National Steel Policy and finally a circular and sustainable steel economy.

## Data Availability

The datasets used and/or analysed during the current study available from the corresponding author on reasonable request.
